# Heat shock response in noise-induced hearing loss: effects of alanyl-glutamine dipeptide supplementation on heat shock proteins status^☆^

**DOI:** 10.1016/j.bjorl.2019.04.012

**Published:** 2019-06-08

**Authors:** Marcos Soares, Analu B. dos Santos, Tainara M. Weich, Gabriela Gomes Mânica, Paulo Ivo Homem de Bittencourt, Mirna Stela Ludwig, Thiago Gomes Heck

**Affiliations:** a Universidade Regional do Noroeste do Estado do Rio Grande do Sul (Unijuí), Departamento de Ciências da Vida, Programa de Pós-Graduação em Atenção Integral à Saúde, Ijuí, RS, Brazil; b Universidade Federal do Rio Grande do Sul, Instituto de Ciências Básicas da Saúde, Departamento de Fisiologia, Porto Alegre, RS, Brazil

**Keywords:** HSP72, Alanyl-glutamine dipeptide, Noise-induced hearing loss, Heat shock response, HSP72, Dipeptídeo de alanil-glutamina, Perda auditiva induzida por ruído, Resposta de choque térmico

## Abstract

**Introduction:**

The 72 kDa heat shock protein, HSP72, located intracellularly provides cochlear cytoprotective and anti-inflammatory roles in the inner ear during stressful noise challenges. The expression of intracellular HSP72 (iHSP72) can be potentiated by alanyl-glutamine dipeptide supplementation. Conversely, these proteins act as pro-inflammatory signals in the extracellular milieu (eHSP72)*.*

**Objective:**

We explore whether noise-induced hearing loss promotes both intracellular and extracellular HSP72 heat shock response alterations, and if alanyl-glutamine dipeptide supplementation could modify heat shock response and prevent hearing loss.

**Methods:**

Female 90 day-old Wistar rats (*n* = 32) were randomly divided into four groups: control, noise-induced hearing loss, treated with alanyl-glutamine dipeptide and noise-induced hearing loss plus alanyl-glutamine dipeptide. Auditory brainstem responses were evaluated before noise exposure (124 dB SPL for 2 h) and 14 days after. Cochlea, nuclear cochlear complex and plasma samples were collected for the measurement of intracellular HSP72 and extracellular HSP72 by a high-sensitivity ELISA kit.

**Results:**

We found an increase in both iHSP72 and eHSP72 levels in the noise-induced hearing loss group, which was alleviated by alanyl-glutamine dipeptide treatment. Furthermore, H-index of HSP72 (plasma/cochlea eHSP72/iHSP72 ratio) was increased in the noise-induced hearing loss group, but prevented by alanyl-glutamine dipeptide treatment, although alanyl-glutamine dipeptide had no effect on auditory threshold.

**Conclusions:**

Our data indicates that cochlear damage induced by noise exposure is accompanied by local and systemic heat shock response markers. Also, alanyl-glutamine reduced stress markers even though it had no effect on noise-induced hearing loss. Finally, plasma levels of 72 kDa heat shock proteins can be used as a biomarker of auditory stress after noise exposure.

## Resumo

**Introdução:**

A proteína de choque térmico de 72 kDa, HSP72 localizada intracelularmente (iHSP72) possui papéis citoprotetores e anti-inflamatórios cocleares na orelha interna durante situações de ruído estressantes. A expressão dessa proteina pode ser potencializada pela suplementação com dipeptídeo de alanil-glutamina. Por outro lado, essas proteínas atuam como sinais pró-inflamatórios no meio extracelular (eHSP72).

**Objetivo:**

Investigar se a perda auditiva induzida por ruído promove alterações tanto das proteínas HSP72 intracelulares quanto extracelulares na resposta de choque térmico e se a suplementação com alanil-glutamina pode modificar a resposta de choque térmico e evitar a perda auditiva.

**Método:**

Ratos Wistar fêmeos, com 90 dias de idade (n = 32) foram divididas aleatoriamente em quatro grupos: controle, perda auditiva induzida por ruído, tratados com alanil-glutamina e perda auditiva induzida por ruído mais alanil-glutamina. Os potenciais evocados auditivos do tronco encefálico foram avaliados antes da exposição ao ruído (124 dB NPS por 2 h) e 14 dias após. A cóclea, o complexo nuclear coclear e amostras de plasma foram coletadas para mensuração de HSP72 intra e extracelular com um kit Elisa de alta sensibilidade.

**Resultados:**

Houve um aumento nos níveis de HSP72 intra e extracelular no grupo perda auditiva induzida por ruído, que foi minimizado pelo tratamento com alanil-glutamina. Além disso, o índice H das HSP72 (razão eHSP72 no plasmacom alanil-glutamina, embora/cóclea) aumentou no grupo perda auditiva induzida por ruído, mas foi limitado pelo tratamento com alanil-glutamina, embora o alanil-glutamina não tenha efeito no limiar auditivo.

**Conclusões:**

Nossos dados indicam que o dano coclear induzido pela exposição ao ruído é acompanhado por marcadores da resposta de choque térmico locais e sistêmicos. Além disso, alanil-glutamina reduziu os marcadores de estresse, mesmo não tendo efeito sobre a perda auditiva induzida por ruído. Finalmente, os níveis plasmáticos de proteínas de choque térmico de 72 kDa podem ser usados como biomarcador do estresse auditivo após a exposição ao ruído.

## Introduction

Hearing loss affects approximately 360 million people worldwide, with a great impact on relationships and the ability to communicate. Although noise is the main etiological risk factor for hearing damage, it is estimated that 10% of the human population is exposed to excessive sound pressure, at levels that may induce auditory injury. Noise-Induced Hearing Loss (NIHL) is the most prevalent occupational disease in the US, with 22 million workers exposed to high levels of noise, requiring close to 240 million dollars in hearing loss treatment.^1^, ^2^, ^3^ Many therapeutic strategies to treat or prevent NIHL have been investigated. Antioxidant therapies have shown success in preventing oxidative stress induced by noise exposition in animal models.^3^ The Food and Drug Administration has recommended investigations into orally administered alternatives for hearing diseases.^4^, ^5^

A high level of noise exposure promotes intense metabolic activity in the cochlea, which induces oxidative stress associated with transient or permanent cochlear hair cell damage.^4^, ^6^, ^7^ For protection against noise challenges, the cochlea requires a cytoprotective response in the form of the expression of a family of 70 kDa Heat Shock Proteins (HSP70). HSPA1A is the most studied Heat Shock Response (HSR) gene, due to its high expression in mammalian cells under stress conditions. It is located at the Major Histocompatibility Complex (MHC) III region and encodes a 72 kDa inducible form (HSP72). In studies of heat-shocked preconditioned mice, the expression of these proteins increased, which provided protection against noise-induced hearing damage; this highlights the importance of HSP70 expression.^8^ Also, suppression of Heat Shock Factor 1 (HSF1), the transcription factor required for HSP72 synthesis, was shown to result in permanent hearing loss after noise exposition.^9^

Several studies have assessed methods to induce the HSR in cochlea by assessing the cytoprotective role of HSP70.^10^, ^11^, ^12^, ^13^, ^14^ Glutamine is an amino acid that has been evaluated as a HSR potentiator, in both in vitro and in vivo studies.^15^, ^16^ The influence of glutamine or alanyl-glutamine Dipeptide (DIP) on intracellular antioxidant defense due to increased glutathione levels,^17^ and the presence of glutamine transporters in cochlear hair cells, suggests that glutamine supplementation may be important for auditory health in subjects exposed to noise.^18^

Intracellular HSP72 (iHSP72) acts as a molecular chaperone of other proteins (thereby limiting protein aggregation, facilitating protein refolding and maintaining structural function), and has anti-inflammatory properties through the inhibition of nuclear factor kB (NF-kB) activation.^19^ On the other hand, an increasing number of observations indicate that when located in extracellular *milieu* (eHSP72), these proteins can affect adjacent or distant cells.^20^, ^21^ eHSP72 is able to promote molecular interactions with cell surface receptors, and thus promote pro-inflammatory cell-signaling by interaction with a variety of eHSP72-receptors. In this respect, the release of eHSP72 to the extracellular *milieu* can be characterized as a pro-inflammatory state, while intracellular expression of iHSP72 represents a broader anti-inflammatory role. Based on these observations, the eHSP72/iHSP72 ratio (H-index) was established, where [eHSP72/iHSP70] at basal state is 1:1 = 1 (i.e., control group) represents a normal condition.^22^, ^23^, ^24^ To our knowledge, the H-index has never been used in hearing loss studies. In the present study using rats, we investigated whether noise exposition could induce HSR locally in the cochlea (iHSP72) and systemically (eHSP72). We also assessed whether DIP supplementation coulg modify HSR and prevent hearing loss. We hypothesized that plasma eHSP72 levels and/or the H-index can be used as biomarkers of auditory stress after noise exposure.

## Methods

### Animals

Female 90 day-old Wistar rats (*n* = 32) weighing approx. 200 g were obtained from (Animal Facility of Regional University of the Northwestern Rio Grande do Sul State – (UNIJUÍ). They were maintained under a controlled temperature (23 ± 1 °C) in a 12/12 h light/dark cycle (lights on at 07:00 a.m.), and housed in plastic cages (49 × 34 × 16 cm). Throughout the experiments, the rats had access to water and were fed with standard pelleted laboratory chow (Nuvilab®) ad libitum. The absence of otitis after specialist otoscopy was used as some inclusion criteria. Animals were randomly assigned into groups as described in the “Experimental design” section. The investigation followed all ethical rules established by Brazilian Guidelines for the Care and Use of Laboratory Animals and the Guide for Care and Use of Laboratory Animals. and the Guide for Care and Use of Experimental Animals, published by the National Institutes of Health (NIH publication n° 85–23, revised in 1996). All procedures were approved by the Committee of Animal Welfare (CEUA-UNIJUI, protocol n° 058/15). All experimental procedures were made between October 2016 and February 2017. Biochemical analysis was made between March and June of 2017.

### Experimental design

**Figure 1:**
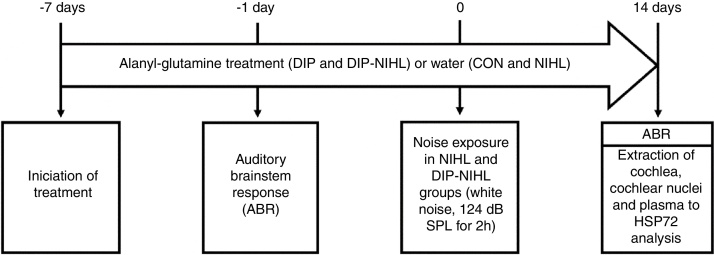
Experimental design. Seven days before the induction of the animal model of noise-induced hearing loss, treatment with alanyl-glutamine (DIP and DIP-NIHL groups) or water (CON and NIHL groups) was started. One day before, auditory evaluation with ABR was performed to determine basal hearing thresholds. At day zero, the NIHL and DIP-NIHL groups were exposed to 124 dB SPL for 2 h. After 14 days, ABR was performed in all animals, followed by extraction of the cochlea, cochlear nuclei, and plasma for measurement of HSP72 concentration.

For seven consecutive days, the rats received oral alanyl-glutamine Dipeptide (DIP) or water (vehicle), and were then evaluated by the Auditory Brainstem Response test (ABR). One day later, half of the animals were exposed to noise for 2 h and then all (*n* = 32) were split into the following experimental groups (*n* = 8 per group): Control (CON), Noise-Induced Hearing Loss (NIHL), treated with DIP (DIP) and NIHL plus DIP treatment (DIP + NIHL). The DIP and DIP + NIHL groups were treated with DIP for more than fourteen days after noise exposition. The ABR test was repeated in all rats, and cochlea and nuclear cochlear complexes were surgery extracted by a specialist. Plasma samples were also obtained for analyses. A summary of the experimental design is shown in Fig. 1.

### Alanyl-glutamine dipeptide (DIP) supplementation

The rats were supplemented daily with l-alanyl-l-glutamine DIP (Dipeptiven®, Fresenius Kabi®) at a dose of 1.5 g/kg (diluted in water to a final concentration of 0.2 g/mL). The animals received supplements through gavage feeding (1 mL/100 g bodyweight) for 21 days (7 days before and 14 days after, noise exposition).

### Auditory brainstem response (ABR)

For the auditory evaluation of the rats, Auditory Brainstem Response (ABR) was performed using the Vivosonic Integrity V500 system®. The rats were anesthetized intraperitoneally with ketamine (80 mg/kg) and xylazine (10 mg/kg), and placed in an anechoic room. Subcutaneous needle-type electrodes were inserted posterior to the tested pinna (active electrode), vertex (reference electrode) and contralateral pinna (ground electrode). The sound stimuli were clicks (rise/fall time, 2 ms; total duration, 2 ms; repetition rate, 21 s). The responses were filtered (100–3000 Hz bandpass), and averaged across 500 samples.

The rat ABR consists of four components (labeled P1–P4) which occur within 6 ms of the stimulus onset. These components reflect the neural activity of the auditory nerve (P1), the cochlear nucleus (P2), the superior olivary complex (P3), and the lateral lemniscus and/or inferior colliculus (P4). Hearing thresholds were determined by decreasing the sound intensity in 5 dB steps, starting at 100 dB and decreasing to 0 dB, or until a reliably-scored ABR component was detected. In rodents, the ABR P2 wave is the largest and usually the last to disappear as the sound stimulus decreases. Hence, the threshold value was defined as the lowest intensity able to elicit a P2 wave,^25^ and ABR data was expressed as Hearing Thresholds (HT) and Hearing Threshold Shifts (HTS). The latter represents the difference between the thresholds, before and after the noise exposure of each rat.

### Noise exposure

The rats were exposed for 2 h to continuous white noise of a broad spectrum of frequency and a peak intensity of 8000 Hz, at 124 dB SPL. During the exposure, the rats were placed in a box inside an anechoic room. White noise was produced by an audio signal generator, connected to speakers in the center of the box (EP125, Insight®). The noise level was measured using a decibelimeter (TDEC100 Digital Decibelimeter, Incoterm®) located inside the box at the start, and after completion, of the noise exposure.

### Cochlea and cochlear nuclear complex iHSP72 and plasma eHSP72 levels

Cochlea (two for each animal) and nuclear cochlear complex samples were quickly removed and washed in ice-cold Phosphate-Buffered Saline (PBS), pH 7.4. Samples were homogenized mechanically inside microtubes containing 50 μL of potassium phosphate buffer with protease inhibitor (Phenyl-Methyl-Sulfonyl Fluoride, PMSF, 100 μM) and centrifuged (5000 rpm for 10 min). The supernatants were frozen in microtubes containing liquid nitrogen, until analysis of iHSP70 levels. Plasma was obtained by centrifugation (blood with EDTA; 3000 rpm for 10 min) and stored at −20 °C for analysis of eHSP72 levels. Cochlea and nuclear cochlear complex iHSP70 levels and plasma eHSP70 levels were measured using a high sensitivity ELISA kit (AMP’D® HSP70 high sensitivity ELISA kit, EnzoLifesciences®).

### Extracellular-to-intracellular HSP70 ratio index (H-index)

Extracellular-to-intracellular HSP70 ratio index (H-index) has been described as a novel and overall index of the immunoinflammatory status of an individual.^19^, ^22^, ^23^, ^24^, ^26^, ^27^ The rationale for H-index is that the higher the level of eHSP70, the greater the inflammatory signal, due to the pro-inflammatory nature of the protein. Conversely, if cells are able to respond to stressful stimuli by enhancing iHSP70 production, they simultaneously enter a state of anti-inflammation. Therefore, if Rc = [eHSP70]c/[iHSP70]c represents the HSP70 ratio in a controlled situation, the H-index for a stressful situation (Rj) can be calculated as the quotient of different values of Rj = [eHSPA]j/[iHSPA]j, relative to Rc (where Rc = 1, i.e., baseline). Hence, the H-index (Rj/Rc) allows comparisons between any stressful situation and the control.^22^

### Statistical analysis

Preceding the statistical analysis, all outcome variables were assessed for normality using the Kolmogorov–Smirnov test. Data is presented as the mean ± S.D. For the analysis of hearing loss and HSR response, the minimum sample size required to detect differences (keeping *α* = 0.05 and test power of 80%), is 8 rats in each group.^28^ The *t*-test was used to compare basal and final hearing thresholds within groups. Comparisons between groups were performed by one-way ANOVA followed by Student–Newman–Keuls post hoc test.

## Results

**Figure 2:**
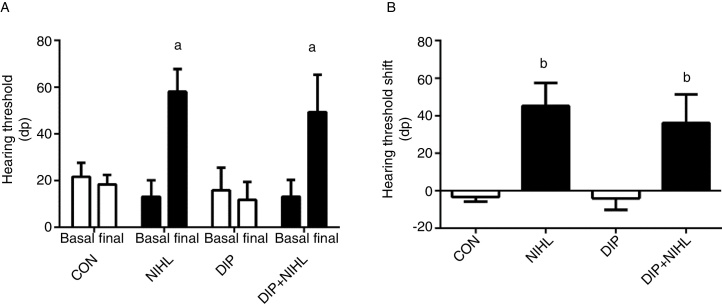
Effects of alanyl-glutamine dipeptide (DIP) treatment on noise-induced hearing loss (NIHL). (A) Hearing threshold and (B) Hearing threshold shift. NIHL and DIP+NIHL showed an increase in hearing threshold (^a^*p* < 0.05 vs. same group before noise exposure) and hearing threshold shift (^b^*p* < 0.05 vs. groups without noise exposure).

Before any intervention, all the rats showed an auditory threshold close to 20 dB (Fig. 2A). Noise exposition promoted a 40 dB increase in auditory threshold in the NIHL and the DIP+NIHL groups (Fig. 2A and B).

**Figure 3:**
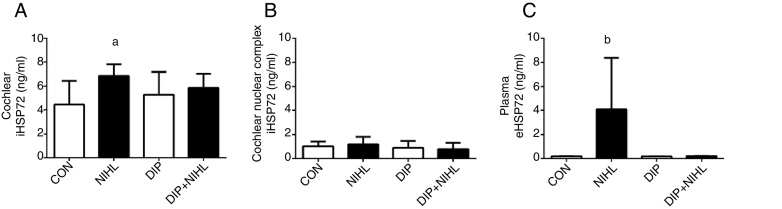
Effects of alanyl-glutamine Dipeptide (DIP) treatment on Noise-Induced Hearing Loss (NIHL) Heat Shock Response (HSR). (A) NIHL promoted an increase in cochlear iHSP72 expression (^a^*p* < 0.05 vs. control). (B) No changes in iHSP72 expression were observed in cochlear nuclear complex. (C) Plasma eHSP72 concentrations were higher in the NIHL group than all groups combined (^b^*p* < 0.05).

Noise exposition (14 days) induced an increase in cochlear iHSP70 levels (NIHL group) when compared to the control group (Fig. 3A), whilst iHSP70 levels in the nuclear cochlear complex were unaltered (Fig. 3B). The eHSP72 levels increased due to noise exposition, and this effect was blunted by DIP treatment (Fig. 3C).

**Figure 4:**
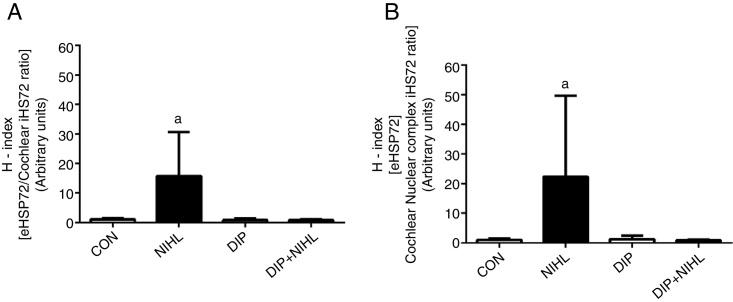
Effects of alanyl-glutamine Dipeptide (DIP) treatment on Noise-Induced Hearing Loss (NIHL) eHSP72/iHSP72 ratio (H-index). (A) plasma/cochlear HSP70 ratio and (B) plasma/nuclear cochlear complex HSP70 ratio. NIHL promoted an increase in both plasma/cochlear and plasma/cochlear nuclear complex eHSP72/iHSP72 ratio (^a^*p* < 0.05 vs. all groups).

After noise exposition, the eHSP70/iHSP70 ratio heat shock status (H-index) was evaluated. Increases were observed in both plasma/cochlear HSP70 ratio (Fig. 4A) and plasma/nuclear cochlear complex HSP70 ratio (Fig. 4B). Treatment with DIP removed these effects and resulted in H-index levels, in plasma/cochlear and plasma/nuclear cochlear complex, that were similar to the control group.

## Discussion

This study reports the first evidence that cochlear damage induced by noise exposure is accompanied by local and systemic HSR, and DIP supplementation can attenuate stress markers in a NIHL rat model. We also observed that DIP treatment did not prevent hearing loss, despite the decreases in stress marker levels.

The rat model of NIHL was based on a previous study, in which loss of hearing was induced using 124 dB SPL for 2 h.^28^ This differed from our study in the use of Sprague–Dawley rats, and peak sound intensity set at a frequency of 4000 Hz. The present study also used a click stimulus for ABR. Both click and pure-tone stimuli are commonly used in investigations of this nature. The click stimulus has high reproducibility and waveform stability, and is one of the most common stimuli used in clinical studies. In humans, auditory evaluation using clicks produces a frequency spectrum of 2000–4000 Hz, compared to 8000–10 000 Hz in rats. A possible explanation for this difference is the anatomical differences of human and rat ears.^29^ For our study, click stimulus was suitable for cochlear evaluation because of its excellent reproducibility. Due to the spectral matching (8000–10 000 Hz) of the click stimulus, a peak sound intensity of 124 dB SPL was chosen at 8000 Hz. Even with the differences between these studies, both sets of results demonstrated similar hearing threshold shifts of approximately 40 dB, 14 days after noise exposure.

Tissue HSR is required for the maintenance of a balanced inflammatory status, due to the cytoprotective and anti-inflammatory roles of iHSP70 expression. This is essential for proteostasis against harmful challenges such as oxidative stress.^30^ Several stressors are able to induce iHSP70 expression in the cochlea of rodents, including whole body heat-shock,^8^ local hyperthermia,^31^ transitory isquemia,^32^ cisplatin ototoxicity^33^ and high levels of noise exposition.^8^, ^10^, ^34^ The role of iHSP70 in situations of noise stress was investigated by Fairfield et al.^9^ and Gong et al.^35^ Both groups showed the importance of Heat Shock Factor 1 (HSF1), the main transcription factor of HSP70 family, in cochlear damage prevention and repair after intense noise exposition in knockout hsf−/− mice.^9^, ^35^ HSR initiated soon after noise exposure (106 dB NPS for 2 h), with a peak of HSP70 mRNA expression after 4 h.^35^ We have demonstrated, for the first time, a persistent stress response of the auditory system after a noise challenge. Rats from the NIHL group exhibited sustained increases in iHSP70 levels for 14 days after noise exposure. However, DIP treatment did not result in potentiation of iHSP70 in cochlear or cochlear nuclear complex and so failed to prevent hearing loss (i.e., the posology dose, frequency and period of treatment) of DIP did not lead to improvements in HSR compared to the control rats. The failure to initiate a robust HSR under stressful situations is a serious impairment of cell function.^22^, ^36^

The InterAcademy Medical Panel recommends the avoidance and treatment of NIHL.^1^ Accordingly, we investigated the effect of DIP supplementation on hearing loss. l-glutamine is the most abundant free amino acid in the body, nutritionally classified as a nonessential amino acid. Since glutamine is the immediate precursor of glutamate, de novo synthesis of glutathione may increase with DIP treatment, and provide an additional antioxidant defense in intracellular spaces^17^, ^37^ and thus prevent oxidative stress damage.^38^ These effects have been observed in studies that administered the same DIP doses (1.5 g/kg) used in our study.^39^, ^40^ Only a few studies have investigated glutamine cochlear metabolism. Ryan and Schwartz^41^ traced glutamine uptake into cochlear hair cells, and observed higher levels in the inner hair cells than those of the outer cells. This suggests the existence of a membrane high-affinity system for glutamine transport in cochlea. In addition, glutamate is the main neurotransmitter used by cochlea auditory signaling, mainly by inner hair cells. Consequently, these cells are rich in glutaminase enzymes.^42^ Dendritic presynaptic membranes express high amounts of α-amino-3-hydroxy-5-methyl-4-isoxazolepropionic acid receptor (AMPA) glutamate receptor SAT1 (alternatively termed GlnT, SA2, SNAT1 and ATA1), a member of the neutral amino acids transporter family (SLC38). It has preferential affinity for glutamine, and transports this amino acid from the endolymph (extracellular space) to inner hair cells for glutamate synthesis.^18^

The protective effects of glutamine are associated with HSR potentiation, as it can promote a slight increase in Heat Shock Factor 1 (HSF-1) trimerization, a step required for HSR. However, under challenge situations such as heat shock, glutamine increases HSF1 activation, and consequently HSP70 synthesis.^37^ Activation of the O-linked glycosylation (O-GlcNAc) pathway by glutamine can promote reciprocal phosphorylation,^15^ which leads to nuclear translocation of HSF1 to the Heat Shock Element (HSE) region that is responsible for HSP70 family expression.^16^

These factors strengthen the hypothesis that DIP supplementation is beneficial to the auditory system, with roles in signaling, antioxidant and cell stress defense. Although DIP supplementation (at 1.5 g/kg) can promote a 62% increase in plasma glutamine concentration,^17^ the DIP dose required to improve cochlear cell function and protection is unclear.^38^ Additionally, the time-course of cochlear HSR under noise stress requires further investigation, especially since rapid HSR was observed after 4 h and maintained for the 14 days of the trial.

A crucial aspect of HSP70 physiology is the versatility and duality of these proteins. iHSP70 acts as anti-inflammatory agent by inhibiting the activation of nuclear transcription factors of the kappa light chain enhancer of activated B cells (κB, family; NF-κB) at multiple regulatory levels.^37^ In contrast, eHSP70 has an opposite function. It signals the presence of homeostatic challenges to physiological systems, after binding to Toll-like receptors (TLR-2, -4 and -7) in a variety of cells.^19^ This leads to the activation of pro-inflammatory pathways.^43^ Since the discovery of eHSP70s in the circulatory system, many associations between eHSP70 levels and bad prognosis in patients have been described in several diseases, usually related to oxidative stress. Release of eHSP70 occurs through several pathways. It is thought that HSP70 is found in plasma as a result of an active process such as danger signaling, or by passive necrotic cell death.^21^ For the first time, we have demonstrated that the harmful effects of noise exposition can be detected by an increase in eHSP72 plasma levels. Furthermore, the decrease in eHSP70 may facilitate the development of DIP-based therapy for the treatment of hearing loss.

We evaluated cochlea and nuclear cochlear complex iHSP70 and plasma eHSP70 levels using a high sensitivity ELISA kit. It was assumed that both the inducible HSPA1A and HSPA6 (HSP70B′) forms, as well as the cognate HSPA8 form of HSP70, would accumulate in the extracellular space of different cell types after appropriate stressful stimuli. However, only the HSPA1A ELISA kit has undergone global evaluation, and has the sensitivity (pg/mL range) to detect minute HSP70 quantities in culture media and sera. Additionally, previous studies in this laboratory^23^ have indicated that the principal eHSP70 forms (HSPA1A and HSPA8) are secreted in similar amounts. Therefore, it was surmised that HSPA1A is representative of the total eHSP70 secretion. The results of the present study indicate that cochlear damage induced by noise exposition is accompanied by local and systemic HSR. Thus, plasma levels of eHSP72 can be used as a biomarker of cochlear stress due to noise exposure.

Due to the versatility of HSP70 in inducing different inflammation responses according to its location, it is likely that this protein may be an important marker for immunoinflammatory state during exercise.^26^ Indeed, its level in circulation appears to be fundamental to the maintenance of homeostasis.^19^ In addition, the [eHSP72/iHSP70] ratio balance, measured by the mathematical calculation H-index, may represent an important biomarker of health, and serve as a reference for subclinical biological processes.

Our data demonstrates that DIP treatment blunts the release of eHSP72 in noise-exposed rats. Possibly, glutamine decreases oxidative stress and thereby reduces cellular stress. We can discard the hypothesis that decreases in eHSP72 release are due to cellular malfunction (e.g., cell death, necrosis or apoptosis), since the auditory threshold was similar in the NIHL and DIP+NIHL groups. In addition, the H-index mean in the NIHL group was 16.0, whilst in the DIP+NIHL group it was approximately 1.0 (arbitrary units of eHSP72/iHSP72 concentration). These findings are satisfactory because H-index may stratify HSP70 status (i.e., H-index of one, relative to the normal profile, and higher H-indices under a stress profile), as well as other clinical conditions according to their immunoinflammatory states.^22^ Indeed, the H-index has recently emerged as a potential biomarker of the effect of stressful situations on the immune system, and of immunoinflammatory imbalances related to cytokine fluctuations and poor HSR. Finally, the high H-Index value observed herein (15.7 in NIHL group) is approaching the inflection point (19.18, arbitrary units of eHSP72/iHSP70 ratio), which represents a dangerous pro-inflammatory profile.^22^

## Conclusion

Our data indicates that cochlear damage induced by noise exposition is accompanied by local and systemic heat shock responses. Also, DIP supplementation did not prevent noise-induced hearing loss, but did promote a reduction in stress markers. The results of this study confirm that plasma levels of 72 kDa heat shock proteins can be used as biomarkers of auditory stress after noise exposure.

## Funding sources

This work was supported by grants from the 10.13039/501100004263Research Support Foundation of the State of Rio Grande do Sul (PqG-2013-FAPERGS process 002106-2551/13-5, and ARD/PPP/FAPERGS/CNPq-08/2014 process 16/2551-0000196-6), and by CNPq (UNIVERSAL MCTI/CNPq N° 01/2016). ABS was a recipient of a scholarship from the Coordination for the Improvement of Higher Education Personnel (CAPES).

## Conflicts of interest

The authors declare no conflicts of interest.
